# Beyond supermarkets: ethnic stores, food environments, and the limits of the Food Access Research Atlas

**DOI:** 10.3389/fpubh.2025.1655436

**Published:** 2025-10-27

**Authors:** Rujia Xie

**Affiliations:** ^1^Trinity College of Arts and Sciences, Duke University, Durham, NC, United States; ^2^Division of Nutritional Sciences, Cornell University, Ithaca, NY, United States

**Keywords:** Food Access Research Atlas, Nutritional Environment Measures Survey, food environment, crowd sourcing, ethnic stores, cultural sensitivity, minority health, health equity

## Abstract

**Background:**

The Food Access Research Atlas (FARA) is a nationally used measure for community food environment that informs resource allocation to improve food access and population health. However, because FARA flags census tracts (CTs) as low-access solely based on CT population’s proximity to supermarkets, it assumes supermarkets as the gold standard of food stores and may not adequately capture the food environments in racial minority neighborhoods, where ethnic stores can play a critical role.

**Objective:**

To examine the accuracy of FARA and its underlying assumption by comparing FARA with our novel Google Maps-based Measure and evaluating the healthfulness of diverse food store types with our multi-ethnic compilation of Nutritional Environment Measures Survey (NEMS).

**Methods:**

This cross-sectional study in Durham, North Carolina, leveraged Google Maps to develop three CT-level variables for food store access (intensity, per capita count, and density) and compared them between low-access and not-low-access CTs classified by FARA. This study then developed the first multi-ethnic NEMS compilation and conducted it among small, large, conventional, and ethnic food stores in Durham to evaluate their respective ability to provide healthy, affordable, and quality food.

**Results:**

The geographic distribution of low-access CTs was not consistent with that of CT-level store count. The intensity, per capita count, and density of large stores and ethnic stores did not significantly differ between low-access and not-low-access CTs. From NEMS, ethnic and large food stores could provide healthier, more affordable, and higher quality food than conventional and small food stores.

**Conclusion:**

By highlighting FARA’s limitations in measuring community food environment and casting doubt on FARA’s underlying assumption, this study highlights the need to shift the discourse away from the binary narrative that a lack of supermarkets equals a food desert, and instead examine the food access provided by existing networks of grocery stores, particularly ethnic stores in minority neighborhoods.

## 1 Introduction

People acquire, prepare, and consume food in a multidimensional context called a “food environment” (1). There are four dimensions of food environments: community, consumer, organizational, and information environments (2). The community environment describes the type, location, and accessibility of food outlets. The consumer environment represents the in-store availability, price, and placement of healthy food options. The organizational environment refers to food sources at schools and worksites, and the information environment is the image and advertising of food being presented (2). This paper will focus on the first two dimensions – community and consumer food environments.

A food desert is a subtype of community food environment in which people have limited access to healthy and affordable food (3, 4). According to the United States Department of Agriculture (USDA), 17.4% (53.6 million) of the United States (US) population currently resides in food deserts (5, 6). This is concerning as living in a food desert is one environmental contributor to an unhealthy diet, which can elevate the risks for heart disease, cancer, stroke, and diabetes – four of the 10 leading causes of death in the US (7–12). Residing in a food desert also correlates with a higher body mass index (BMI) and a higher rate of obesity, increasing the risks for various chronic diseases (13–15). Given the observed correlation between food deserts and chronic health outcomes, the accuracy of community food environment measures becomes imperative to understand the mechanisms underlying the relationship. The Food Access Research Atlas (FARA), developed by the USDA, is one nationally used measure of community food environments at the census tract (CT) level. Census tracts are small, relatively permanent statistical subdivisions of a county defined by the US Census Bureau with a population size between 1,200 and 8,000 (16). FARA categorizes whether a CT is low access (i.e., a food desert) based on the CT population’s proximity to supercenters, supermarkets, and large grocery stores (17). If more than 500 individuals or 33% of the population are beyond 1 mile from the nearest supermarket in an urban CT, or beyond 10 miles in a rural CT, then FARA flags it as a low-access CT (17). FARA’s low-access designation impacts resources allocated to a CT, since only low-access CTs qualify for the USDA’s Healthy Food Financing Initiative, which provides loans, grants, and resources to improve food access (18).

Despite its wide applications in policy space, FARA’s capacity to represent the community food environment in racial minority communities is debatable, as its exclusive focus on supermarkets can overlook small food stores and ethnic markets. Previous research has highlighted the lower supermarket availability in neighborhoods of racial and ethnic minorities (7, 19, 20). However, studies across North Carolina, Maryland, and New York revealed that minority neighborhoods, despite having fewer supermarkets, have twice as many small grocery stores as predominantly White neighborhoods (21). These small food stores, particularly those in minority neighborhoods, can provide critical access to healthful foods (20–24). Small ethnic stores, for example, offer culturally appropriate items that are often absent in mainstream or conventional supermarkets (25–28). Ethnic stores can also enable a better shopping experience with employees who understand minorities’ food practices and/or speak their languages (29). Thus, to examine FARA’s accuracy in describing community food environments, it is important to test whether FARA’s designation of CT’s low-access status overlooks a CT’s access to ethnic stores.

Behind FARA’s equation of limited supermarket access to food deserts is FARA’s assumption on the consumer food environment that large conventional supermarkets are the “gold standard” of food stores, providing nutritious and affordable food that other food stores (e.g., ethnic stores and corner stores) cannot. Therefore, it is also necessary to evaluate FARA’s underlying assumption by comparing the consumer food environment of small, large, conventional, and ethnic stores. In assessing the consumer food environment, previous studies have employed store audits like the Nutritional Environment Measures Survey (NEMS), an internationally used measure developed by Glanz et al. (30, 31). By looking at the presence, price, and quality of a pre-specified list of “healthy food items” (e.g., skim milk and low-fat baked goods), NEMS generates scores reflecting food stores’ availability, affordability, and quality of healthy items. However, one major drawback of NEMS is its lack of culturally appropriate food in its pre-specified scoresheet, which means that having healthy ethnic items (e.g., corn tortillas or brown rice) does not give ethnic stores higher scores in healthy food availability as it should. This limitation introduces bias to the scoring of nutritional values of ethnic stores and hinders meaningful comparisons between ethnic stores and conventional stores. To address the limitation, multiple researchers have adapted NEMS to better reflect the food environment of a specific location or a subtype of food stores (32–38). Although these adaptations have all been validated with high reliability values, each of them is tailored to only one specific store type. Given the diversity of ethnic stores in the US food landscape, there arises a need for a multi-ethnic version of NEMS to ensure inclusive and accurate measurement of food stores when testing the validity of FARA’s foundational assumption on consumer food environments.

To examine the accuracy of FARA and its underlying assumption, this study has two objectives: (1) to develop the Google Maps-based Measure – a CT-level indicator of food store access consisting of three variables – and compare the community food environment it outlined with that by FARA, and (2) to conduct a novel multi-ethnic compilation of NEMS to rate the consumer food environments of small, large, conventional, and ethnic stores. This study hypothesizes that FARA’s classification of low-access CTs does not accurately reflect the absence of access to healthy, affordable, and quality food, as FARA overlooks ethnic stores (community food environment), which can provide such access (consumer food environment).

## 2 Methods

### 2.1 Community food environment: FARA and Google Maps-based Measure

For the first objective, this study derived CT-level variables from Google Maps data to assess CTs’ food store access, and then compared the community environment of Durham, North Carolina (NC), outlined by FARA and the Google Maps-based Measure. This study used the 2019 FARA data (the most recent dataset available) of the 60 CTs in Durham, NC, and collected the low-access status (low-access or not-low-access) for each CT (39). Google Maps was used as a data source for the comparison to FARA due to its widespread use as a crowd-sourced review platform for retail food stores and supermarkets. Its real-time updates, contributed by individuals with internet access, provided information on food store statuses (e.g., operational, temporarily closed, permanently closed). The high accessibility and low cost of the Google Maps dataset compared to other retail store databases also made Google Maps the most cost-effective data source for this study.

The data on all current food stores in Durham, NC, was scraped from Google Maps in August 2023 using food-service-related queries, including “Grocery Store,” “Ethnic Store,” “Food Store,” “Convenience Store,” “Market,” and “Food Shop.” This process yielded a dataset of 335 food-related stores, encompassing their name, category, business status, rating, point location, and URL of the business’s website. Based on these information, 157 stores were excluded, including stores with locations outside of Durham, NC (*n* = 25), store types not qualified for the study (e.g., coffee shops, gift shops, restaurants) (*n* = 122), and store content not matching its label (e.g., Italian restaurants that Google Maps categorized as ethnic stores) (*n* = 10). No stores in the dataset were listed as “permanently closed,” and stores listed as “temporarily closed” were included unless they met one of the exclusion criteria, in case their status had changed. The remaining 178 food stores comprised the final dataset for analysis, including 4 stores listed as “temporarily closed.”

To categorize the food stores based on type, an ethnic store was identified by meeting any of the following criteria: (1) labeled with an ethnic store category on its Google Maps page (e.g., “Korean grocery store,” “Mexican grocery store”) (*n* = 13), (2) having an ethnic name (e.g., Tropicana Supermarket, Mi Vaquita Mini Market) (*n* = 25), or (3) having a name that indicates the presence of international food items (e.g., Leone International Foods, Around The World Market, Li Ming’s Global Mart) (*n* = 4). For stores that only met the third criterion, the ethnic classification was confirmed by manually checking whether images and/or reviews on the stores’ Google Maps page indicated the availability of ethnic food items. This classification method was based on the approaches used in prior studies on ethnic stores, and it yielded a list of 29 ethnic stores in Durham, with some stores meeting more than one criterion (29, 40). All other stores not classified as ethnic were categorized as conventional stores (e.g., Food Lion, Target, Circle K) (*n* = 149).

To categorize food stores by size, a small store was defined as having (1) less than 2,000 square feet, (2) fewer than five aisles, and (3) only one cash register, to align with the definition set by the corner store NEMS adaptation in this study’s multi-ethnic NEMS compilation (34, 41). Due to limited time and tools to measure the square footage of each individual store, this study used the latter two criteria for size determination. All chained supermarkets were grouped as large stores, such as Target, Food Lion, Walmart, Harris Teeter, and Costco, since they all had more than five aisles (*n* = 29). Then, any stores labeled as a “Convenience store,” “Dollar store,” or “Gas station store” on Google Maps were grouped as small stores, since they had fewer than five aisles and only one register (*n* = 99). The remaining stores (*n* = 50) were individually assessed by manually checking the number of aisles and/or cash registers in customer-posted photos on each store’s Google Maps page. If a customer-posted photo showed five or more aisles or two or more cash registers, the store was categorized as a large store; otherwise, a small store. Stores without any image on Google Maps were visited in person to determine the size.

The 178 food stores were systematically categorized into four mutually exclusive groups by size (small or large) and type (conventional or ethnic): (1) small conventional stores, (2) small ethnic stores, (3) large conventional stores, and (4) large ethnic stores. With the point location and grouping of all 178 food stores, this study used ArcGIS Pro 2.9.0 (ESRI, Redlands, CA) and the USA Geocoding Locator to aggregate store counts at the CT level, and then computed three Google Maps-based variables for the four groups of food stores in each CT: intensity, per capita count, and density. Intensity represented the proportion of food stores in a CT relative to the total number of food stores in Durham, NC. Count per capita represented the ratio of food stores in a CT to the total population of that CT, as per the population data from the 2019 American Community Survey 5-year estimates (42). Density was derived from dividing the CT’s total count of food stores by its total land area. These three variables were calculated because intensity, per capita count, and density are commonly used parameters in small area analysis, which examines disparities among well-defined small areas (i.e., the 60 CTs) within a larger geographic boundary (i.e., Durham County, NC) (21, 43). The three Google Maps-based variables were subsequently compared between low-access CTs and not-low-access CTs identified by FARA. If FARA properly measures food access, not-low-access CTs should have significantly higher intensity, per capita count, and density of food stores than low-access CTs.

### 2.2 Consumer food environment: multi-ethnic NEMS-S

For the second objective, this study compiled a multi-ethnic NEMS survey and sampled 50 food stores in Durham, NC, to conduct in-person NEMS store audits, rating the consumer environments of small, large, conventional, and ethnic stores. This study used four versions of NEMS: (1) the original NEMS-S survey designed for large conventional supermarkets, (2) the NEMS-Corner Store (NEMS-CS) survey tailored for small conventional stores, (3) the Latino adaptation (Latino NEMS-S) customized for Latino ethnic stores, and (4) the Chinese adaptation (C-NEMS-S) for Asian ethnic stores (as there was no validated Asian adaptation for NEMS-S at the time of the study) (30, 34–36). Each store was assigned only one of the four NEMS versions for store auditing based on its size and type. Table 1 lists the food categories evaluated in each NEMS version. Across the four versions, 11 to 16 food categories were assessed, with milk, fruits, vegetables, meat or poultry, grains or bread, and beverages being consistently included, reaching a balance between cross-version comparability and cultural responsiveness.

**Table 1:** Content and score ranges of the Nutritional Environment Measures Survey-Store (NEMS-S), NEMS-Corner Store (NEMS-CS), Latino NEMS-S, and Chinese NEMS-S.

	NEMS-S	NEMS-CS	Latino NEMS-S	Chinese NEMS-S
Evaluated food categories				
Milk	X	X	X	X
Eggs			X	
Fruits	X	X	X	X
Frozen & canned fruits		X	X	
Vegetables	X	X	X	X
Frozen & canned vegetables		X	X	
Starchy tubers				X
Ground beef	X	X		
Fresh beefsteak			X	
Fresh/frozen chicken			X	
Meat & poultry				X
Fresh/frozen fish			X	
Canned fish			X	
Seafood				X
Hot dogs	X	X		
Frozen dinners	X	X		
Baked goods	X	X		
Beverages	X	X	X	X
Bread	X	X		X
Baked chips	X	X		
100-calorie snacks		X		
Cereal	X	X		
Tortillas			X	
Instant noodles				X
Canned beans			X	
Dry beans			X	X
Rice			X	
Grains				X
Cooking oils			X	
Dietary oils				X
Total number of categories	11	14	16	12
Possible range of raw scores				
Availability	[0, 30]	[0, 37]	[0, 46]	[0, 42]
Affordability	[−9, 18]	[−9, 18]	[−7, 14]	[−12, 24]
Quality	[0, 6]	[0, 6]	[0, 6]	[0, 9]
Total score	[−9, 54]	[−9, 61]	[−7, 66]	[−12, 75]

Three subscores were generated from each NEMS: availability, affordability, and quality (44). Table 1 shows the possible score range of each version, and Supplementary material A contains the detailed scoring sheet of all four versions. The availability subscore assessed the presence (and absence) of the pre-specified list of healthy food items. The minimum possible score for availability represented the absence of any healthy food items (e.g., skim milk, whole grain bread), and the maximum represented the presence of a diverse array of healthy food items (e.g., the presence of ≥10 varieties of fresh fruits earned 3 points, 1–5 varieties earned 1 point, and the absence of fresh fruits earned 0 points). The affordability subscore compared the lowest unit price of a food item’s regular and healthier options. The minimum score for affordability indicated that for all applicable food items, the healthy option was less affordable than the regular option, whereas the maximum indicated that the healthy option was more affordable for all applicable food items. Noted, affordability was tied to the availability of healthy food items within the store. When a healthier option was present, the raw subscore was −1 if the healthier option was pricier than the regular counterpart, and 2 if the opposite held true. However, when a healthier option was not available, the raw affordability subscore was 0, since a price comparison could not be made. Consequently, stores lacking healthier options might receive a higher affordability subscore than those offering healthier (but more expensive) options. The quality subscore reflected the freshness and quality of fruits and vegetables. The minimum score for quality indicated that there were no fruits or vegetables (or, in the Chinese NEMS, fruits, vegetables, or seafood) at all in the store, or that only less than a quarter of the fresh produce had an acceptable quality. The maximum represented the presence of all fresh produce, with over three-quarters of them meeting acceptable quality standards. Like affordability, the quality subscore was contingent upon the presence of fresh produce within the store, as the intention behind the quality subscore was to “award” additional points to stores with acceptable quality fresh produce. Therefore, stores without any fresh produce received a quality subscore of 0, and stores with acceptable fruits (i.e., maxing out the quality subscore for fruits) but no vegetables (i.e., no points allocated for vegetables) received half of the maximum possible value. The total NEMS score was calculated as the simple sum of these three subscores. Since the availability subscore contributed the greatest number of points to the total score among the three subscores, it carried the greatest weight, followed by affordability and quality.

The four NEMS versions had different ranges for raw scores. To ensure comparability across stores audited with different versions, each store’s raw scores were standardized to a 0–100 scale using min-max transformation. For each score, the minimum possible raw value for that version (which would be a negative value for the affordability subscore across all four versions, for example) was rescaled to 0, the maximum possible raw value to 100, and all values in between were converted proportionally using the formula X_scaled=100×(X−X_min)/(X_max−X_min) . The total score was first calculated as the simple sum of the three raw subscores and then standardized. Therefore, the subscores and total score were all on a 0–100 scale, and the relative weighting of subscores (availability, affordability, and quality from the highest to lowest) was preserved in the standardized total score.

To conduct the multi-ethnic NEMS, a sample of 50 stores was selected, and in-person visits were carried out between September and October 2023. The sample size was determined by feasibility considerations, as all data collection and analysis were conducted by a single researcher within a limited time frame. Within this constraint, stratified random sampling was used to ensure representation across food store groups (small, large, conventional, and ethnic), selecting a predetermined number of stores in each group using a computer-based random number generator. Due to the much smaller number of ethnic stores compared to conventional stores in Durham, NC, ethnic stores were intentionally oversampled to enable meaningful comparisons. If large conventional stores significantly outperform other store groups in NEMS scores, then FARA’s sole focus on supermarket access in its designation of low-access CTs may be justified. Otherwise, FARA’s assumption underlying its designation of low-access CTs is implausible, which supports the hypothesis.

### 2.3 Statistical analysis

The comparison among CTs and among food stores used the Wilcoxon rank-sum test, given the non-normal distribution of the variables, to detect any statistically significant differences. R 4.3.1 (The R Foundation for Statistical Computing, Vienna, Austria) was used for statistical analyses. *p* < 0.05 was used to determine statistical significance.

## 3 Results

### 3.1 Community food environment: FARA and Google Maps-based Measure

Of the 60 CTs in Durham, NC, 55 were urban, applying the 1-mile distance threshold for low-access CT designation, and 5 were rural, applying the 10-mile threshold. Table 2 presents the descriptive statistics for FARA’s designation of Durham CTs, count of all food stores in Durham, NC, by size and type, and Google Maps-based Measure across CTs. Nearly half (48.3%) of the Durham CTs were low-access CTs. Among the 178 food stores in Durham, there were 31 large conventional stores, 118 small conventional, 10 large ethnic, and 19 small ethnic. Based on the median values for Google Maps-based variables, each Durham CT had 1.69% of the county’s total food stores, 4.20 stores per 10,000 population, and 0.95 stores per square mile of land area.

**Table 2:** Descriptive statistics of the Food Access Research Atlas (FARA), count of all food stores in Durham, NC, by size and type, and Google Maps-based variables.

	*N*	%
FARA’s designation of CTs’ low-access status		
Low-Access CTs	29	48.3
Not-Low-Access CTs	31	51.7
Food stores in Durham, NC, by size and type		
Large Conventional Stores	31	17.4
Small Conventional Stores	118	66.3
Large Ethnic Stores	10	5.6
Small Ethnic Stores	19	10.7
	Median	IQR
Google Maps-based variables across CTs		
Intensity	1.69	[0.56, 2.25]
*Per Capita* Count	4.20	[1.57, 9.15]
Density	0.95	[0.15, 2.63]

CT, census tract; IQR, interquartile range; Intensity, percent of food stores in the CT relative to county total; Per Capita Count, number of food stores in the CT per 10,000 population; Density, number of food stores in the CT per square mile land area.

**Figure 1:**
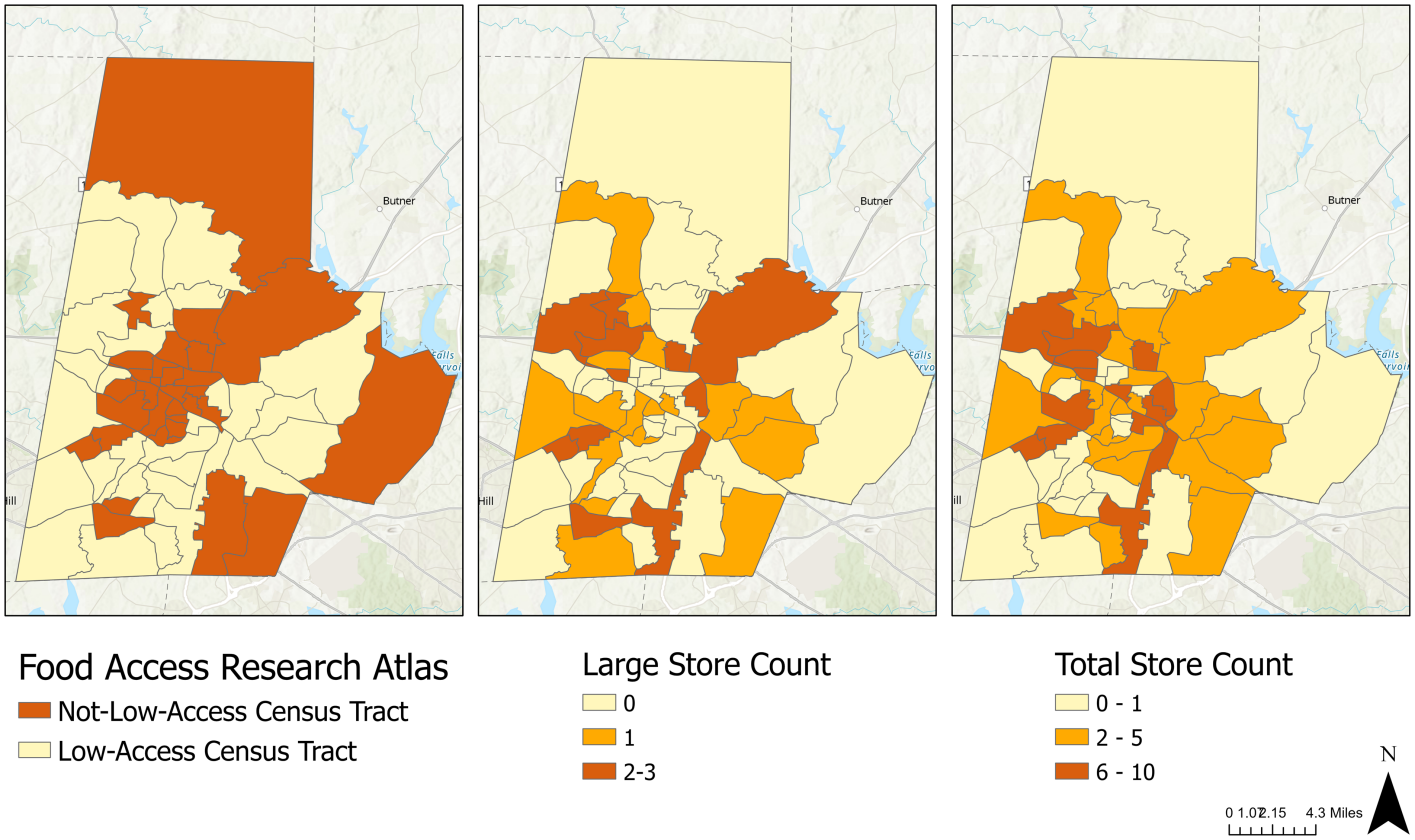
Geographic distribution of low-access census tracts (left), large stores (middle), and total stores (right) in Durham, NC.

To visually compare community food environments described by FARA and Google Maps, Figure 1 displays the geographic distribution of low-access and not-low-access CTs from FARA (left), the CT count of large food stores in Durham, NC, from Google Maps (middle), and the CT count of total food stores (right). Across the three maps in Figure 1, CTs without food access, either labeled by FARA as low-access CTs or having minimal food stores, were marked in light yellow. CTs with food access, either labeled by FARA as not-low-access CTs or having more food stores, were marked in orange or dark red. If FARA accurately captures CTs’ access to food stores, the three maps in Figure 1 should exhibit a consistent color pattern, signifying that FARA’s low-access CTs indeed have limited access to food stores. However, Figure 1 indicates inconsistencies. FARA’s low-access CTs were predominantly clustered in western, southern, and eastern Durham. Meanwhile, clusters of food stores, particularly large stores, appeared in similar areas – western, southern, and eastern Durham. Additionally, the northernmost and easternmost CTs of Durham, both categorized as not-low-access CTs by FARA, had few food stores of any type. This discrepancy could be due to FARA’s default setting designating CTs without data as not-low-access CTs. Consequently, community food environments outlined by FARA and Google Maps-based Measure diverged: some of FARA’s low-access CTs exhibited clusters of large food stores, while certain not-low-access CTs lacked food stores.

In addition to store count, Table 3 compares FARA’s low-access CTs with not-low-access CTs in their Google Maps-based variables – intensity, per capita count, and density of food stores. For intensity, which is the percent of food stores in a CT relative to the county total, no significant differences between low-access and not-low-access CTs were observed for any store group, even large food stores that were accounted for in FARA. For per capita count, not-low-access CTs had significantly more total, small, and conventional food stores per 10,000 population compared to low-access CTs, but no significant difference was found for large and ethnic stores. Similarly, for density, which is the number of food stores per square mile of land area, there was no significant difference in large and ethnic food stores between low-access and not-low-access CTs. To account for potential challenges related to sample size, estimates of differences were examined in addition to *p*-values. Compared to low-access CTs, not-low-access CTs had 1.4 more large stores per 10,000 population, 0.11 more large stores per square mile, and the same number of ethnic stores per 10,000 population and per square mile – which were insufficient to establish significant gaps. In other words, low-access CTs did not have significantly fewer supermarkets or ethnic stores than their not-low-access counterparts.

**Table 3:** Intensity, per capita count, and density of food stores in all, low-access, and not-low-access census tracts (CTs).

Google Maps-based variables	All CTs (*n* = 60)	Low-access CTs (*n* = 29)	Not-low-access CTs (*n* = 31)	*p* value
	Median [IQR]	Median [IQR]	Median [IQR]	
Intensity				
Total stores	1.69 [0.56, 2.25]	1.12 [0.56, 2.25]	1.69 [0.56, 3.09]	0.29
Large stores	0.00 [0.00, 2.44]	0.00 [0.00, 2.44]	2.44 [0.00, 2.44]	0.33
Small stores	1.46 [0.00, 2.37]	0.73 [0.00, 2.19]	1.46 [0.36, 2.92]	0.40
Conventional stores	1.34 [0.67, 2.68]	0.67 [0.67, 2.68]	2.01 [0.34, 2.68]	0.45
Ethnic stores	0.00 [0.00, 3.45]	0.00 [0.00, 0.00]	0.00 [0.00, 3.45]	0.21
*Per Capita* count				
Total stores	4.20 [1.57, 9.15]	2.61 [1.48, 5.86]	7.46 [2.97, 12.68]	0.01
Large stores	0.00 [0.00, 2.52]	0.00 [0.00, 1.34]	1.40 [0.00, 3.37]	0.06
Small stores	3.96 [0.62, 7.68]	1.97 [0.00, 4.27]	5.05 [2.03, 10.53]	0.03
Conventional stores	3.48 [1.41, 6.91]	1.98 [1.34, 4.73]	5.92 [2.62, 11.91]	0.03
Ethnic stores	0.00 [0.00, 1.61]	0.00 [0.00, 0.00]	0.00 [0.00, 2.71]	0.09
Density				
Total stores	0.95 [0.15, 2.63]	0.44 [0.13, 0.98]	1.98 [0.29, 5.08]	0.02
Large stores	0.00 [0.00, 0.66]	0.00 [0.00, 0.28]	0.11 [0.00, 1.22]	0.09
Small stores	0.65 [0.00, 2.54]	0.43 [0.00, 0.94]	1.70 [0.06, 4.24]	0.03
Conventional stores	0.59 [0.12, 2.26]	0.43 [0.13, 0.78]	1.72 [0.08, 3.51]	0.05
Ethnic stores	0.00 [0.00, 0.29]	0.00 [0.00, 0.00]	0.00 [0.00, 1.00]	0.10

IQR, interquartile range; Intensity, percent of food stores in the CT relative to county total; Per Capita Count, number of food stores in the CT per 10,000 population; Density, number of food stores in the CT per square mile land area.

To further explore the lack of association between not-low-access CTs and the number of large stores, this study conducted an additional analysis separating large stores into large conventional stores and large ethnic stores. Table 4 shows that none of the observed differences between low-access and not-low-access CTs reached statistical significance. Therefore, not-low-access CTs did not have a significantly higher intensity, per capita count, or density of total large stores, large conventional stores, or large ethnic stores than low-access CTs.

**Table 4:** Intensity, per capita count, and density of large conventional stores and large ethnic stores in all, low-access, and not-low-access census tracts (CTs).

Google Maps-based variables	All CTs (*n* = 60)	Low-access CTs (*n* = 29)	Not-low-access CTs (*n* = 31)	*p* value
	Median [IQR]	Median [IQR]	Median [IQR]	
Intensity				
Large conventional stores	0.00 [0.00, 3.23]	0.00 [0.00, 3.23]	0.00 [0.00, 3.23]	0.51
Large ethnic stores	0.00 [0.00, 0.00]	0.00 [0.00, 0.00]	0.00 [0.00, 0.00]	0.54
*Per Capita* count				
Large conventional stores	0.00 [0.00, 1.44]	0.00 [0.00, 1.23]	0.00 [0.00, 3.01]	0.25
Large ethnic stores	0.00 [0.00, 0.00]	0.00 [0.00, 0.00]	0.00 [0.00, 0.00]	0.44
Density				
Large conventional stores	0.00 [0.00, 0.28]	0.00 [0.00, 0.16]	0.00 [0.00, 0.81]	0.29
Large ethnic stores	0.00 [0.00, 0.00]	0.00 [0.00, 0.00]	0.00 [0.00, 0.00]	0.50

IQR, interquartile range; Intensity, percent of food stores in the CT relative to county total; Per Capita Count, number of food stores in the CT per 10,000 population; Density, number of food stores in the CT per square mile land area.

### 3.2 Consumer food environment: multi-ethnic NEMS-S

Table 5 provides the count of food stores sampled for the NEMS store audit, stores with completed NEMS data, and the difference between the two. During the fieldwork, one conventional store was reclassified from large to small after an in-person visit revealed it was smaller than indicated on Google Maps. One large ethnic store and two small ethnic stores were found permanently closed during in-person visits, resulting in three fewer ethnic stores than initially planned.

**Table 5:** Number of food stores in Durham, NC, sampled for the Nutritional Environment Measures Survey (NEMS) store audit, completed the audit, and the difference between the two.

Food store group	Sampled	Audited	Difference
Large conventional stores	10	9	−1
Small conventional stores	15	16	+1
Large ethnic stores	10	9	−1
Small ethnic stores	15	13	−2
Total	50	47	−3

To evaluate and compare the consumer food environments of small, large, conventional, and ethnic stores, Table 6 presents the summary statistics of the standardized NEMS total score and subscores. The median standardized total NEMS score was 45.98 among the 47 audited stores, and the stores scored the highest on quality (100,00), followed by availability (54.35) and affordability (33.33). Large ethnic stores had the highest median in NEMS total score, healthy food availability, and healthy food affordability, and shared the highest median in fresh produce quality with large conventional stores.

**Table 6:** Summary statistics of the standardized Nutritional Environment Measures Survey (NEMS) scores of audited stores by store size and type.

NEMS score	All stores	Large conventional stores	Large ethnic stores	Small conventional stores	Small ethnic stores
	(*n* = 47)	(*n* = 9)	(*n* = 9)	(*n* = 16)	(*n* = 13)
	Median [IQR]	Median [IQR]	Median [IQR]	Median [IQR]	Median [IQR]
Total Score	45.98 [32.86, 68.25]	68.25 [66.67, 71.43]	73.97 [69.86, 76.71]	29.29 [25.36, 32.86]	44.83 [41.10, 48.28]
Subscore					
Availability	54.35 [31.08, 86.67]	86.67 [86.67, 93.33]	86.96 [82.61, 91.30]	25.68 [18.92, 30.41]	52.17 [42.86, 54.76]
Affordability	33.33 [33.33, 37.57]	37.04 [33.33, 40.74]	38.10 [30.56, 47.62]	33.33 [33.33, 37.04]	33.33 [33.33, 33.33]
Quality	100.00 [16.67, 100.00]	100.00 [100.00, 100.00]	100.00 [100.00, 100.00]	25.00 [0.00, 50.00]	66.67 [0.00, 100.00]

IQR, interquartile range.

**Figure 2:**
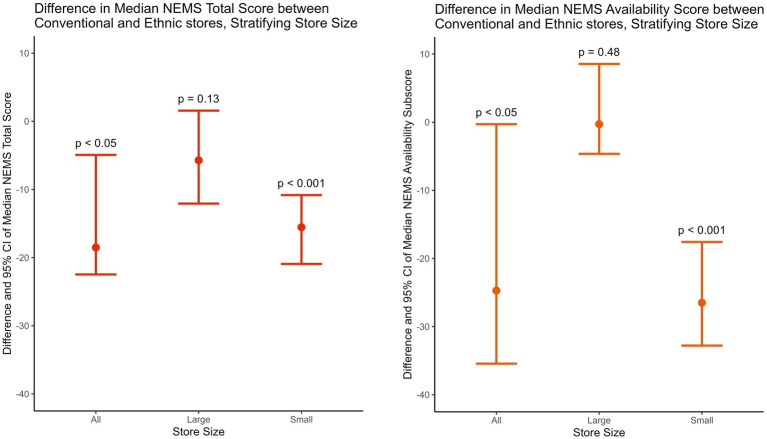
The difference between conventional and ethnic stores in the Nutritional Environment Measures Survey’s median total and availability scores and their 95% confidence intervals (CIs), stratified by store size.

The study then examined how the size and type of food store individually impacted its NEMS scores. Table 7 presents the 95% confidence intervals (CIs) of the difference between conventional and ethnic stores in NEMS scores, stratified by store size. Figure 2 zooms in to visualize the difference in NEMS total and availability scores, given that availability carried the greatest weight. Among all stores and small stores, ethnic stores had significantly higher total and availability scores than conventional stores, while no significant difference was found between large conventional and large ethnic stores. In other words, small ethnic stores provided healthier, more affordable, and higher quality food than small conventional stores, while large conventional stores and large ethnic stores demonstrated comparable abilities to provide healthy, affordable, and quality food.

**Table 7:** The difference between conventional and ethnic stores in the Nutritional Environment Measures Survey’s scores and their 95% confidence intervals (CIs), stratified by store size.

NEMS score	Conventional vs. Ethnic stores		Large conventional vs. Large ethnic stores		Small conventional vs. Small ethnic stores	
	95% CI	*p* value	95% CI	*p* value	95% CI	*p* value
Total	[−22.47, −4.93]	<0.05	[−12.07, 1.57]	0.13	[−20.92, −10.82]	<0.001
Availability	[−35.43, −0.29]	<0.05	[−4.64, 8.55]	0.48	[−32.78, −17.57]	<0.001
Affordability	[−3.70, 3.70]	0.96	[−14.29, 21.16]	0.93	[−3.70, 3.70]	0.92
Quality	[−50.00, 0.00]	0.23	-	-	[−50.00, 0.00]	0.19

**Figure 3:**
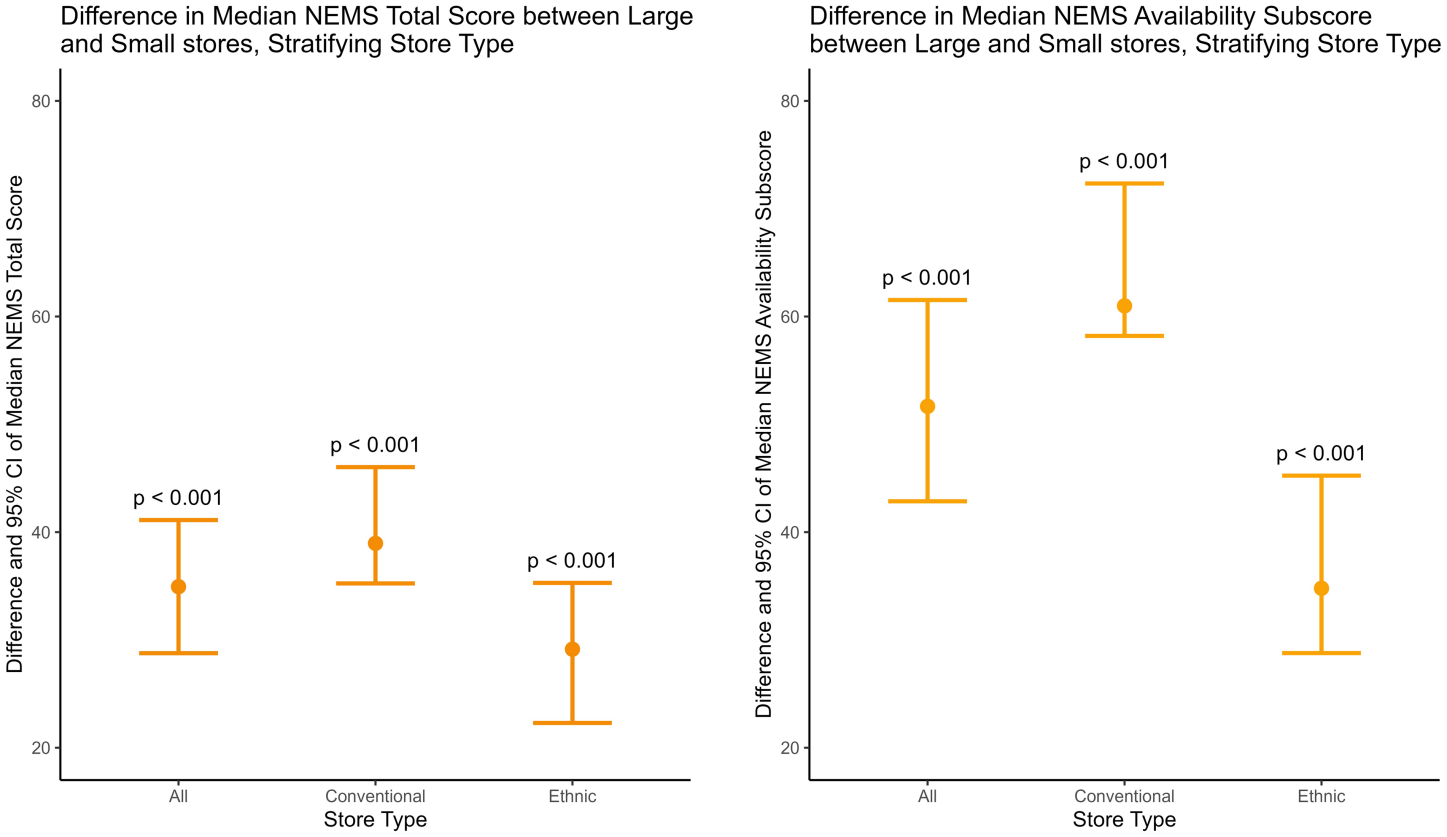
The difference between large and small stores in the Nutritional Environment Measures Survey’s median total and availability scores and their 95% confidence intervals (CIs), stratified by store type.

Stratified by store type, Table 8 shows the 95% CI of the difference between large and small stores in NEMS scores, and Figure 3 visualizes the difference in NEMS total and availability scores. Across store types, large stores had significantly higher total, availability, and quality scores than small stores, while no significant difference was found in the affordability score. In other words, store size was significantly positively associated with a store’s ability to provide healthy and quality food, but it was not associated with healthy food affordability.

**Table 8:** The difference between large and small stores in the Nutritional Environment Measures Survey’s scores and their 95% confidence intervals (CIs), stratified by store type.

NEMS score	Large vs. Small stores		Large conventional vs. Small conventional stores		Large ethnic vs. Small ethnic stores	
	95% CI	*p* value	95% CI	*p* value	95% CI	*p* value
Total	[28.77, 41.12]	<0.001	[35.24, 46.03]	<0.001	[22.30, 35.29]	<0.001
Availability	[42.86, 61.53]	<0.001	[58.20, 72.34]	<0.001	[28.78, 45.24]	<0.001
Affordability	[0.00, 7.41]	0.12	[0.00, 7.41]	0.12	[−9.52, 14.29]	0.62
Quality	[33.33, 100.00]	<0.001	[50.00, 100.00]	<0.001	[0.00, 100.00]	<0.01

## 4 Discussion

This study examined the accuracy of FARA and its underlying assumption by developing the Google Maps-based Measure to assess CT-level food store access, comparing Google Maps-based variables between low-access and not-low-access CTs in Durham, NC, labeled by FARA, and conducting a novel multi-ethnic compilation of NEMS to evaluate the consumer food environments of small, large, conventional, and ethnic stores in Durham. The results substantiated the hypothesis that FARA inadequately represents access to healthy, affordable, and quality food. Two key findings supported this conclusion. First, the discrepancies between Google Maps variables and FARA highlighted FARA’s inaccuracy in depicting the community food environment and its oversight of ethnic stores, as the geographic distribution of low-access CTs was not consistent with that of CT-level store count, and there was no significant difference in the intensity, per capita count, and density of large and ethnic stores between low-access and not-low-access CTs. Second, the NEMS scores revealed that ethnic stores offer better access to healthy, affordable, and quality food than their conventional counterparts.

These findings are significant because, as low-access and not-low-access CTs did not significantly differ in their intensity, per capita count, and density of large stores, this study raises questions about FARA’s definition of supermarkets that future studies can more closely examine, such as whether FARA includes large ethnic stores as supermarkets and how often the FARA supermarket dataset or inventory gets updated. The results also highlight FARA’s limitations in measuring access to ethnic stores, which suggests FARA’s oversight of ethnic stores and potential misrepresentation of food access in minority communities, especially with the prominent roles of ethnic stores in racial minority neighborhoods. Since FARA provides critical guidance for community planning, resource allocation, and scholarly research, this study highlights the need for more culturally responsive food environment measures to enhance the effectiveness, sustainability, and equity of food policies. As one step to close the gap, this study develops the Google Maps-based Measure and is the first to utilize a multi-ethnic compilation of NEMS, serving as a prototype for future refinements and replications.

Importantly, this study showcases the nutritional contributions of ethnic stores and casts doubt on FARA’s assumption that only large conventional supermarkets can offer nutritious and affordable food. In other words, FARA’s exclusive emphasis on supermarkets compromises its accuracy. In addition to FARA, multiple studies have considered supermarkets as the gold standard under the belief that conventional supermarkets have “better availability and selection, higher quality, and lower cost of foods,” which prompted them to focus on attracting supermarkets to minority neighborhoods in order to address racial disparities in food deserts (19, 45). For example, initiatives like the Healthy Food Financing Initiatives have allocated funding to open food outlets in underserved areas, primarily focusing on “full-service grocery stores” (46, 47). Interventions have also been implemented nationwide to introduce supermarkets to racial minority communities (48–52). However, as this study has shown, such an approach carries the risk of overlooking the nutritional contributions of ethnic stores and neglecting the established network and assets within the community. By indicating the nutritional significance of ethnic stores, the results of this study could guide future research on the role of ethnic stores in population health and inform interventions that leverage existing community assets in minority neighborhoods.

The study has a few limitations. First, the ethnic NEMS-S adaptations may not represent the heterogeneity of subgroups within the ethnicity, as the accuracy of the Chinese NEMS-S may differ between Chinese and Korean stores. The absence of price tags was observed in many small ethnic and conventional stores, which can impact the accuracy of the affordability subscore. Also, as shown in the fieldwork, Google Maps’ data on a store’s business status may not accurately reflect the on-the-ground realities. While field verification of the sample’s status through in-person observation could improve the accuracy, it was beyond the time frame and capacity of this study. In addition, although stratified sampling and oversampling of ethnic stores were used to ensure representation, the number of stores within each group (*n* = 9–16) was smaller than ideal due to the relatively small number of ethnic stores in Durham, NC, and feasibility constraints. With limited statistical power, the Wilcoxon rank-sum tests may not have detected smaller but meaningful differences between groups. As the sample size did not proportionally reflect the distribution of food stores across CTs, the sample’s representativeness at the CT level may be compromised. In terms of CTs, the majority of Durham CTs are small in geographic size given the county’s urban context, so our findings may not generalize to rural areas or areas with a significantly higher ratio of rural to urban CTs. However, Durham’s population is socioeconomically and racially diverse, so our results can still shed light on food access among a heterogeneous population (53). Another limitation is the potential temporal mismatch between data sources: the most recent FARA data is dated to 2019, whereas Google Maps and NEMS data were collected in 2023. Changes in store openings and closures during this period may affect comparability. This gap underscores the need for more frequent updates to the FARA dataset to ensure its continued relevance for research and policy. Lastly, unlike small and large conventional stores, which were scored with separate versions (NEMS-S and NEMS-CS), ethnic NEMS adaptations lack distinctions between small and large stores. This means that small ethnic stores were held to the same scoring scale as large ones, while small conventional stores, with NEMS-CS accounting for their limited store space by counting canned items, were held to a lower scoring standard than large conventional stores. This can underestimate the nutritional value of small ethnic stores.

By highlighting the limitations of FARA and disclosing the multi-dimensional and complex nature of the community and consumer food landscape, this paper reveals the need to shift the discourse away from the binary narrative that a lack of supermarkets equals a food desert. This study calls for more attention to asset-based interventions and policies that focus on and strengthen food access provided by existing networks of grocery stores, especially in minority neighborhoods.

## Data Availability

The raw data supporting the conclusions of this article will be made available by the authors, without undue reservation.

## References

[1] Alliance EPH. (2022). What are “food environments”? Available online at: https://epha.org/what-are-food-environments/ [Accessed April 20, 2022]

[2] Glanz K Sallis JF Saelens BE Frank LD. Healthy nutrition environments: concepts and measures. Am J Health Promot. (2005) 19:330–3. doi: 10.4278/0890-1171-19.5.330, PMID: 15895534

[3] Beaulac J Kristjansson E Cummins S. A systematic review of food deserts, 1966−2007. Prev Chronic Dis. (2009) 6:A105. Available online at: http://www.cdc.gov/pcd/issues/2009/jul/08_0163.htm (Accessed April 5, 2022) 19527577 PMC2722409

[4] Walker RE Keane CR Burke JG. Disparities and access to healthy food in the United States: a review of food deserts literature. Health Place. (2010) 16:876–84. doi: 10.1016/j.healthplace.2010.04.013, PMID: 20462784

[5] Dutko P Ploeg MV Farrigan T. (2022). Characteristics and influential factors of food deserts. Available online at: http://www.ers.usda.gov/publications/pub-details/?pubid=45017 [Accessed April 6, 2022].

[6] USDA. (2019) USDA ERS - Documentation. Available online at: https://www.ers.usda.gov/data-products/food-access-research-atlas/documentation/ [Accessed April 6, 2022]

[7] Larson NI Story MT Nelson MC. Neighborhood environments: disparities in access to healthy foods in the U.S. Am J Prev Med. (2009) 36:e10:74–81. doi: 10.1016/j.amepre.2008.09.025 18977112

[8] Mozaffarian D Wilson PWF Kannel WB. Beyond established and novel risk factors. Circulation. (2008) 117:3031–8. doi: 10.1161/CIRCULATIONAHA.107.738732, PMID: 18541753

[9] Grosso G Bella F Godos J Sciacca S Del Rio D Ray S. Possible role of diet in cancer: systematic review and multiple meta-analyses of dietary patterns, lifestyle factors, and cancer risk. Nutr Rev. (2017) 75:405–19. doi: 10.1093/nutrit/nux012, PMID: 28969358

[10] Kontogianni MD Panagiotakos DB. Dietary patterns and stroke: a systematic review and re-meta-analysis. Maturitas. (2014) 79:41–7. doi: 10.1016/j.maturitas.2014.06.014, PMID: 25042875

[11] Jayedi A Soltani S Abdolshahi A Shab-Bidar S. Healthy and unhealthy dietary patterns and the risk of chronic disease: an umbrella review of meta-analyses of prospective cohort studies. Br J Nutr. (2020) 124:1133–44. doi: 10.1017/S0007114520002330, PMID: 32600500

[12] CDC. (2022) National center for health statistics. FastStats. Available online at: https://www.cdc.gov/nchs/fastats/leading-causes-of-death.htm [Accessed April 6, 2022]

[13] Beydoun MA Powell LM Chen X Wang Y. Food prices are associated with dietary quality, fast food consumption, and body mass index among U.S. children and adolescents. J Nutr. (2011) 141:304–11. doi: 10.3945/jn.110.132613 21178080 PMC3021450

[14] Moore LV Diez Roux AV Nettleton JA Jacobs DR Franco M. Fast-food consumption, diet quality, and neighborhood exposure to fast food: the multi-ethnic study of atherosclerosis. Am J Epidemiol. (2009) 170:29–36. doi: 10.1093/aje/kwp090, PMID: 19429879 PMC2733038

[15] Bodor JN Rose D Farley TA Swalm C Scott SK. Neighbourhood fruit and vegetable availability and consumption: the role of small food stores in an urban environment. Public Health Nutr. (2008) 11:413–20. doi: 10.1017/S1368980007000493, PMID: 17617930

[16] US Census Bureau. (2025). Glossary. Available online at: https://www.census.gov/programs-surveys/geography/about/glossary.html [Accessed September 20, 2025]

[17] USDA. (2019). USDA ERS- about the atlas. Available online at: https://www.ers.usda.gov/data-products/food-access-research-atlas/about-the-atlas/ [Accessed April 5, 2022]

[18] USDA. (2021) Rural development: healthy food financing initiative. Available online at: https://www.rd.usda.gov/about-rd/initiatives/healthy-food-financing-initiative [Accessed November 17, 2023]

[19] Zenk SN Schulz AJ Israel BA James SA Bao S Wilson ML. Neighborhood racial composition, neighborhood poverty, and the spatial accessibility of supermarkets in metropolitan Detroit. Am J Public Health. (2005) 95:660–7. doi: 10.2105/AJPH.2004.042150, PMID: 15798127 PMC1449238

[20] Powell LM Slater S Mirtcheva D Bao Y Chaloupka FJ. Food store availability and neighborhood characteristics in the United States. Prev Med. (2007) 44:189–95. doi: 10.1016/j.ypmed.2006.08.008, PMID: 16997358

[21] Moore LV Diez Roux AV. Associations of neighborhood characteristics with the location and type of food stores. Am J Public Health. (2006) 96:325–31. doi: 10.2105/AJPH.2004.058040, PMID: 16380567 PMC1470485

[22] Bower KM Thorpe RJ Rohde C Gaskin DJ. The intersection of neighborhood racial segregation, poverty, and urbanicity and its impact on food store availability in the United States. Prev Med. (2014) 58:33–9. doi: 10.1016/j.ypmed.2013.10.010, PMID: 24161713 PMC3970577

[23] Galvez MP Morland K Raines C Kobil J Siskind J Godbold J. Race and food store availability in an inner-city neighbourhood. Public Health Nutr. (2008) 11:624–31. doi: 10.1017/S1368980007001097, PMID: 17935646

[24] Raja S Ma C Yadav P. Beyond food deserts: measuring and mapping racial disparities in neighborhood food environments. J Plann Educ Res. (2008) 27:469–82. doi: 10.1177/0739456X08317461

[25] Ayala GX Baquero B Klinger S. A systematic review of the relationship between acculturation and diet among Latinos in the United States: implications for future research. J Am Diet Assoc. (2008) 108:1330–44. doi: 10.1016/j.jada.2008.05.009, PMID: 18656573 PMC3727241

[26] Grigsby-Toussaint DS Zenk SN Odoms-Young A Ruggiero L Moise I. Availability of commonly consumed and culturally specific fruits and vegetables in African-American and Latino neighborhoods. J Am Diet Assoc. (2010) 110:746–52. doi: 10.1016/j.jada.2010.02.008, PMID: 20430136 PMC3427830

[27] Wang L Lo L. Immigrant grocery-shopping behavior: ethnic identity versus accessibility. Environ Plan A. (2007) 39:684–99. doi: 10.1068/a3833

[28] Yeh MC Ickes SB Lowenstein LM Shuval K Ammerman AS Farris R. Understanding barriers and facilitators of fruit and vegetable consumption among a diverse multi-ethnic population in the USA. Health Promot Int. (2008) 23:42–51. doi: 10.1093/heapro/dam044, PMID: 18182418

[29] Joassart-Marcelli P Rossiter JS Bosco FJ. Ethnic markets and community food security in an urban “food desert.”. Environ Plan A. (2017) 49:1642–63. doi: 10.1177/0308518X17700394

[30] Glanz K Sallis JF Saelens BE Frank LD. Nutrition environment measures survey in stores (NEMS-S): development and evaluation. Am J Prev Med. (2007) 32:282–9. doi: 10.1016/j.amepre.2006.12.019, PMID: 17383559

[31] McKinnon RA Reedy J Morrissette MA Lytle LA Yaroch AL. Measures of the food environment: a compilation of the literature, 1990–2007. Am J Prev Med. (2009) 36:S124–33. doi: 10.1016/j.amepre.2009.01.012, PMID: 19285203

[32] Franco M Diez Roux AV Glass TA Caballero B Brancati FL. Neighborhood characteristics and availability of healthy foods in Baltimore. Am J Prev Med. (2008) 35:561–7. doi: 10.1016/j.amepre.2008.07.003, PMID: 18842389 PMC4348113

[33] Granfeldt G Victoriano M Carrasco JA Sáez K Bibiloni M d M Tur JA. Adaption and reliability of the nutrition environment measures for stores (NEMS-S) instrument for use in urban areas of Chile. BMC Public Health. (2022) 22:224. doi: 10.1186/s12889-022-12651-w, PMID: 35114954 PMC8815185

[34] Cavanaugh E Mallya G Brensinger C Tierney A Glanz K. Nutrition environments in corner stores in Philadelphia. Prev Med. (2013) 56:149–51. doi: 10.1016/j.ypmed.2012.12.007, PMID: 23262362

[35] Baier JL Palmer SM Winham DM Shelley MC. Development of a nutrition environment assessment tool for Latino ethnic stores. Int J Environ Res Public Health. (2022) 19:1860. doi: 10.3390/ijerph19031860, PMID: 35162882 PMC8834718

[36] Liu Y Song S Gittelsohn J Jiang N Hu J Ma Y. Adaptation and validation of the Chinese version of the nutrition environment measurement tool for stores. Int J Environ Res Public Health. (2019) 16:782. doi: 10.3390/ijerph16050782, PMID: 30836654 PMC6427157

[37] Lo BK Minaker LM Mah CL Cook B. Development and testing of the Toronto nutrition environment measures survey–store (ToNEMS-S). J Nutr Educ Behav. (2016) 48:723–729.e1. doi: 10.1016/j.jneb.2016.07.020, PMID: 27575848

[38] Shaver ER Sadler RC Hill AB Bell K Ray M Choy-Shin J. The Flint food store survey: combining spatial analysis with a modified nutrition environment measures survey in stores (NEMS-S) to measure the community and consumer nutrition environments. Public Health Nutr. (2018) 21:1474–85. doi: 10.1017/S1368980017003950, PMID: 29361993 PMC6941423

[39] USDA. (2019). USDA ERS- go to the atlas. Available online at: https://www.ers.usda.gov/data-products/food-access-research-atlas/go-to-the-atlas/ (Accessed April 5, 2022).

[40] Largent P. (2012) Cross-cultural food consumption in Chicago: the impact of ethnic grocery stores on the availability of a healthy, affordable, and quality food supply. Available online at: https://via.library.depaul.edu/etd/122 (Accessed April 5, 2022).

[41] Healthy Corner Store Initiatives. (2018) Program: healthy corner stores. Available online at: https://foodcommunitybenefit.noharm.org/resources/implementation-strategy/program-healthy-corner-stores (Accessed October 28, 2023).

[42] US Census Bureau. (2019) DP05: ACS demographic and housing estimates. Available online at: https://data.census.gov/table/ACSDP5Y2019.DP05?q=population&g=050XX00US37063$1400000&moe=false&tp=true (Accessed October 15, 2023).

[43] Goldstein B Gounaridis D Newell JP. The carbon footprint of household energy use in the United States. Proc Natl Acad Sci. (2020) 117:19122–30. doi: 10.1073/pnas.1922205117, PMID: 32690718 PMC7431053

[44] Penn NEMS. (2022) Tools. Available online at: https://nems-upenn.org/tools/ [Accessed April 5, 2022]

[45] Pothukuchi K. Attracting supermarkets to inner-city neighborhoods: economic development outside the box. Econ Dev Q. (2005) 19:232–44. doi: 10.1177/0891242404273517

[46] Fleischhacker SE Flournoy R Moore LV. Meaningful, measurable, and manageable approaches to evaluating healthy food financing initiatives: an overview of resources and approaches. J Public Health Manag Pract. (2013) 19:541–9. doi: 10.1097/PHH.0b013e318271c6eb, PMID: 23073081

[47] Healthy Food Finance Initiative. (2022) Impact: America’s healthy food finance initiative. Available online at: https://www.investinginfood.com/impact/. [Accessed April 6, 2022]

[48] Elbel B Mijanovich T Kiszko K Abrams C Cantor J Dixon LB. The introduction of a supermarket via tax-credits in a low-income area: the influence on purchasing and consumption. Am J Health Promot. (2017) 31:59–66. doi: 10.4278/ajhp.150217-QUAN-733, PMID: 26389982

[49] Ghosh-Dastidar M Hunter G Collins RL Zenk SN Cummins S Beckman R. Does opening a supermarket in a food desert change the food environment? Health Place. (2017) 46:249–56. doi: 10.1016/j.healthplace.2017.06.002, PMID: 28648926 PMC5588682

[50] Richardson AS Ghosh-Dastidar M Beckman R Flórez KR DeSantis A Collins RL. Can the introduction of a full-service supermarket in a food desert improve residents’ economic status and health? Ann Epidemiol. (2017) 27:771–6. doi: 10.1016/j.annepidem.2017.10.011, PMID: 29198367 PMC5989716

[51] Rogus S Athens J Cantor J Elbel B. Measuring Micro-level effects of a new supermarket: do residents within 0.5 mile have improved dietary behaviors? J Acad Nutr Diet. (2018) 118:1037–46. doi: 10.1016/j.jand.2017.06.360 28797794

[52] Sadler RC Gilliland JA Arku G. A food retail-based intervention on food security and consumption. Int J Environ Res Public Health. (2013) 10:3325–46. doi: 10.3390/ijerph10083325, PMID: 23921626 PMC3774441

[53] Data USA. (2023) Durham County, NC. Available online at: https://datausa.io/profile/geo/durham-county-nc [Accessed September 21, 2025]

