# Supplementary material for: Beyond supermarkets: ethnic stores, food environments, and the limits of the Food Access Research Atlas
Source: Front Public Health. 2025 Oct 27;13:1655436. doi: 10.3389/fpubh.2025.1655436 (PMC12597754; doi:10.3389/fpubh.2025.1655436)
Supplement: Supplementary file 1 [file Table_1.docx]

**Supplementary Material A: The Scoring Sheets of the Multi-ethnic Nutritional Environment Measures Survey (NEMS) Compilation**

This compilation consists of four prior adaptations of NEMS: 1) NEMS-S for large conventional stores, 2) NEMS-Corner Store (NEMS-CS) for small conventional stores, 3) Latino NEMS-S for Latino ethnic stores, and 4) Chinese NEMS-S (C-NEMS-S) for Asian ethnic stores (due to the lack of validated Asian NEMS-S). The scoring sheet of each version is shown below.

1. NEMS-S

Source: Glanz K, Sallis JF, Saelens BE, Frank LD. Nutrition Environment Measures Survey in Stores (NEMS-S): Development and Evaluation. American Journal of Preventive Medicine. 2007 Apr 1;32(4):282–9.


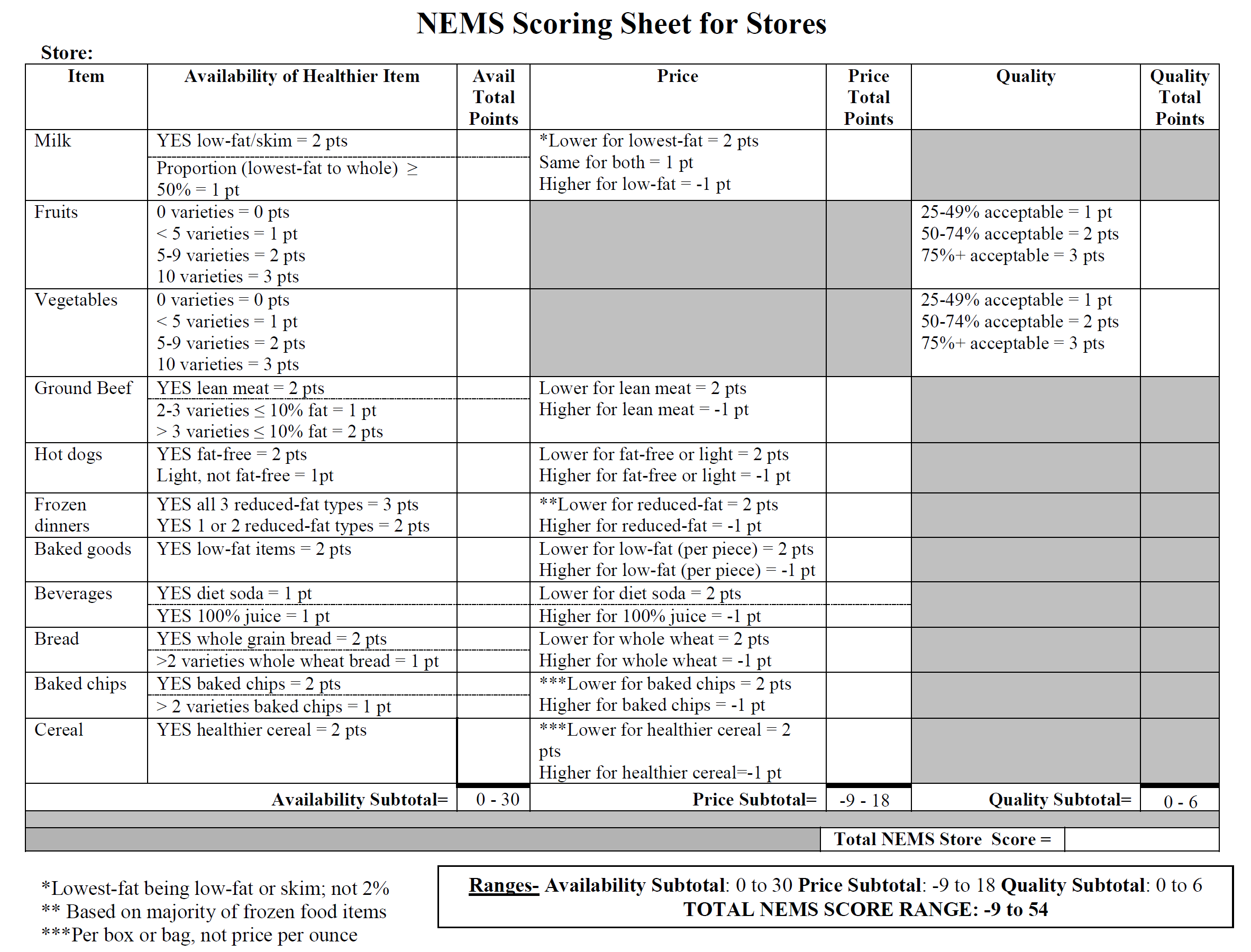
Scoring sheet for NEMS-S:

1. NEMS-Corner Store (NEMS-CS)

Source: Cavanaugh E, Mallya G, Brensinger C, Tierney A, Glanz K. Nutrition environments in corner stores in Philadelphia. Preventive Medicine. 2013 Feb 1;56(2):149–51.

Scoring sheet for NEMS-CS:

1. Latino NEMS-S

Source: Baier JL, Palmer SM, Winham DM, Shelley MC. Development of a Nutrition Environment Assessment Tool for Latino Ethnic Stores. Int J Environ Res Public Health. 2022 Feb 7;19(3):1860.

Scoring sheet for Latino NEMS-S:


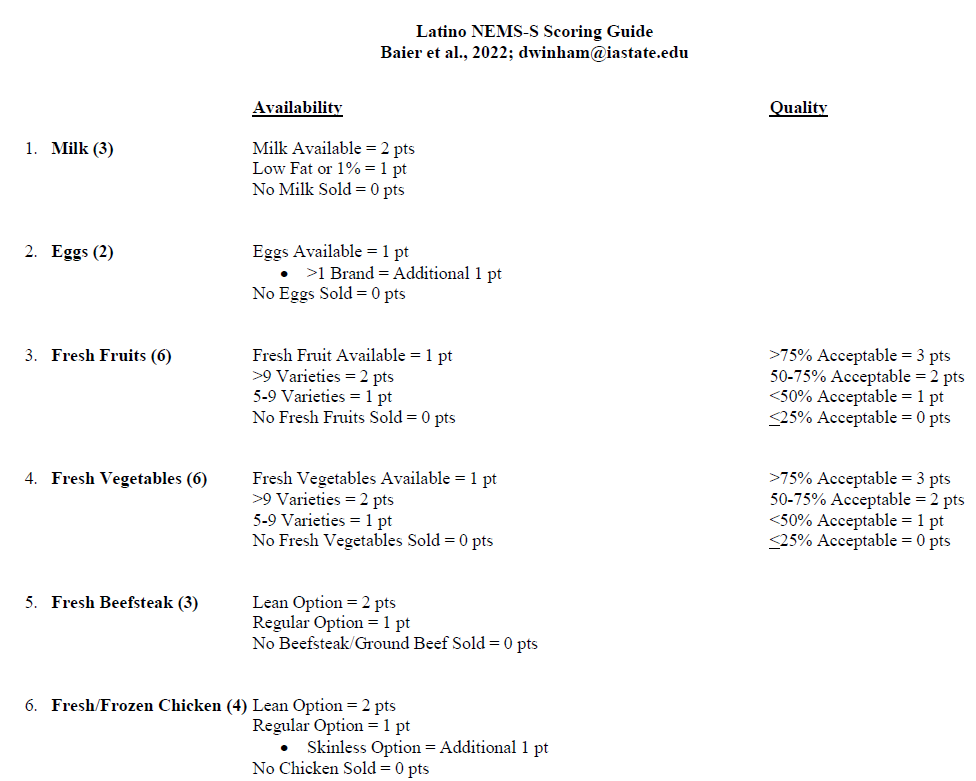


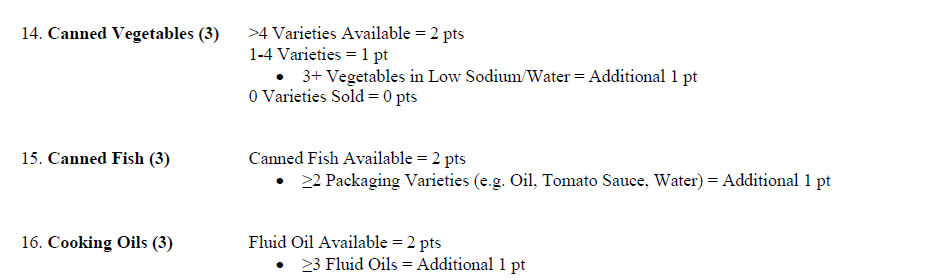

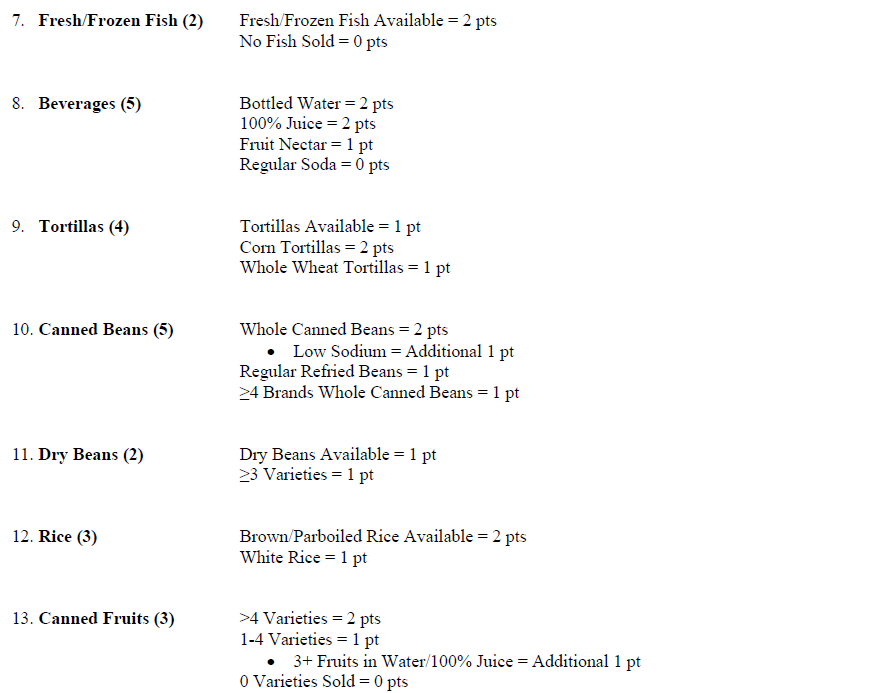


1. Chinese NEMS-S (C-NEMS-S)

Source: Liu Y, Song S, Gittelsohn J, Jiang N, Hu J, Ma Y, et al. Adaptation and Validation of the Chinese Version of the Nutrition Environment Measurement Tool for Stores. International Journal of Environmental Research and Public Health. 2019 Jan;16(5):782.

Scoring sheet for C-NEMS-S:

| **Item** | **Availability of Healthier Option** | **Price** | **Quality** |
| --- | --- | --- | --- |
| Grains | YES whole grains = 2 pts | Lower for whole grains = 2 pts | Not Applicable |
|  | 2–3 species = 1 pt | Higher for whole grains = −1 pt |  |
|  | >3 species = 2 pts |  |  |
| Dry Beans | 0 specie = 0 pt | Not Applicable | Not Applicable |
|  | 1 species = 1 pt |  |  |
|  | 2–3 species = 2 pts |  |  |
|  | ≥4 species = 3 pts |  |  |
| Starchy Tubers | 0 specie = 0 pt | Not Applicable | Not Applicable |
|  | 1 species = 1 pt |  |  |
|  | 2–4 species = 2 pts |  |  |
|  | 5 species = 3 pts |  |  |
| Vegetables | 0 specie = 0 pt | Not Applicable | 25–49% acceptable = 1 pt |
|  | 1–4 species = 1 pt |  | 50–74% acceptable = 2 pts |
|  | 5–9 species = 2 pts |  | 75% + acceptable = 3 pts |
|  | ≥10 species = 3 pts |  |  |
| Fruits | 0 specie = 0 pt | Not Applicable | 25–49% acceptable = 1 pt |
|  | 1–4 species = 1 pt |  | 50–74% acceptable = 2 pts |
|  | 5–9 species = 2 pts |  | 75% + acceptable = 3 pts |
|  | ≥10 species = 3 pts |  |  |
| Seafood | 0 specie = 0 pt | Not Applicable | 25–49% acceptable = 1 pt |
|  | 1–4 species = 1 pt |  | 50–74% acceptable = 2 pts |
|  | 5–9 species = 2 pts |  | 75% + acceptable = 3 pts |
|  | ≥10 species = 3 pts |  |  |
| Meat and Poultry | YES meat with less than 10% fat = 2 pts | Lower for lean meat = 2 pts | Not Applicable |
|  | 2–3 species = 1 pt | Higher for lean meat = −1 pt |  |
|  | >3 species = 2 pts |  |  |
| Dietary oils | YES plant-based oils = 2 pts | Lower for plant-based oils = 2 pts | Not Applicable |
|  | 2–3 species = 1 pt | Higher for plant-based oils = −1 pt |  |
|  | >3 species = 2 pts |  |  |
| Milk | YES low-fat milk = 2 pts | Lower for low-fat milk = 2 pts | Not Applicable |
|  | Proportion (lowest fat to whole) >50% = 1 pt | Same for both = 1 pt |  |
|  |  | Higher for low-fat milk = −1 pt |  |
| Bread | YES whole wheat bread = 2 pts | Lower for whole wheat bread = 2 pts | Not Applicable |
|  | >2 species = 1 pt | Higher for whole wheat bread = −1 pt |  |
| Instant Noodles | YES non-fried instant noodles = 2 pts | Lower for non-fried instant noodles = 2 pts | Not Applicable |
|  | >2 species = 1 pt | Higher for non-fried instant noodles = −1 pt |  |
| Beverages | YES sugar-free carbonated beverages = 1 pt | Lower for sugar-free carbonated beverages = 2 pts | Not Applicable |
|  | YES sugar-free lactose beverages = 1 pt | Higher for sugar-free carbonated beverages = −1 pt |  |
|  | YES sugar-free fruit juice = 1 pt | Lower for sugar-free lactose beverages = 2 pts |  |
|  | YES sugar-free tea beverages = 1 pt | Higher for sugar-free lactose beverages = −1 pt |  |
|  | YES sugar-free vegetable protein beverages = 1 pt | Lower for sugar-free fruit juice = 2 pts |  |
|  | YES sugar-free apple cider vinegar = 1 pt | Higher for sugar-free fruit juice = −1 pt |  |
|  |  | Lower for sugar-free tea beverages = 2 pts |  |
|  |  | Higher for sugar-free tea beverages = −1 pt |  |
|  |  | Lower for sugar-free vegetable protein beverages = 2 pts |  |
|  |  | Higher for sugar-free vegetable protein beverages = −1 pt |  |
|  |  | Lower for sugar-free apple cider vinegar = 2 pts |  |
|  |  | Higher for sugar-free apple cider vinegar = −1 pt |  |
|  | Availability Subtotal = 0 to 42 | Price Subtotal = −12 to 24 | Quality Subtotal = 0 to 9 |
| Total Chinese version of the Nutritional Environment Measurement Survey (C-NEMS) Stores Score = −12 to 75 | | | |

POINT RANGES: Availability Subtotal: 0 to 48; Price Subtotal: −14 to 26; Quality Subtotal: 0 to 9; TOTAL NEMS SCORE RANGE: from −14 to 83.
